# Infra-Population and -Community Dynamics of the Parasites *Nosema apis* and *Nosema ceranae*, and Consequences for Honey Bee (*Apis mellifera*) Hosts

**DOI:** 10.1371/journal.pone.0099465

**Published:** 2014-07-02

**Authors:** Geoffrey R. Williams, Dave Shutler, Karen L. Burgher-MacLellan, Richard E. L. Rogers

**Affiliations:** 1 Department of Biology, Acadia University, Wolfville, Nova Scotia, Canada; 2 Department of Biology, Dalhousie University, Halifax, Nova Scotia, Canada; 3 Institute of Bee Health, Vetsuisse Faculty, University of Bern, Bern, Switzerland; 4 Agroscope, Swiss Bee Research Centre, Bern, Switzerland; 5 Atlantic Food and Horticulture Research Centre, Agriculture and Agri-Food Canada. Kentville, Nova Scotia, Canada; 6 Wildwood Labs, Kentville, Nova Scotia, Canada; University of North Carolina Greensboro, United States of America

## Abstract

*Nosema* spp. fungal gut parasites are among myriad possible explanations for contemporary increased mortality of western honey bees (*Apis mellifera*, hereafter honey bee) in many regions of the world. Invasive *Nosema ceranae* is particularly worrisome because some evidence suggests it has greater virulence than its congener *N. apis*. *N. ceranae* appears to have recently switched hosts from Asian honey bees (*Apis cerana*) and now has a nearly global distribution in honey bees, apparently displacing *N. apis*. We examined parasite reproduction and effects of *N. apis*, *N. ceranae*, and mixed *Nosema* infections on honey bee hosts in laboratory experiments. Both infection intensity and honey bee mortality were significantly greater for *N. ceranae* than for *N. apis* or mixed infections; mixed infection resulted in mortality similar to *N. apis* parasitism and reduced spore intensity, possibly due to inter-specific competition. This is the first long-term laboratory study to demonstrate lethal consequences of *N. apis* and *N. ceranae* and mixed *Nosema* parasitism in honey bees, and suggests that differences in reproduction and intra-host competition may explain apparent heterogeneous exclusion of the historic parasite by the invasive species.

## Introduction

Western honey bees (*Apis mellifera*, hereafter honey bees) are among the most vital and versatile pollinators, contributing to production of 39 of the world's 57 most important crops [1]. Unfortunately, today's beekeepers face significant hurdles to maintain healthy colonies that are capable of crop pollination because of dramatic honey bee colony mortalities in many regions of the world. A great deal of attention has focussed on these mortalities because humanity's reliance on pollinator-dependent crops has increased significantly in the last half century [2]. Honey bee mortality is believed to result from multiple stressors acting alone or in combination, including nutritional deficiencies, management issues, agro-chemicals, and especially introduced parasites [3]–[5].

Significant interest has recently focussed on the newly detected microsporidian gut parasite *Nosema ceranae* because unusually high honey bee colony mortality coincided with its apparent host-switch from Asian honey bees (*Apis cerana*) to honey bees [6], [7], as well as its subsequent widespread dispersal [8]–[12]. *N. ceranae* can cause tissue damage [13]–[15], nutritional stress [16]–[18], and suppression of host immunity [19]. In Spain, *N. ceranae* is typically associated with reduced colony survivorship [20], whereas in other parts of Europe [21] and in North America [22]–[25], its virulence is debated. Possible explanations for this variation include parasite or host genetics [15], [26]–[28], climate [29], [30], nutrition [18], or interactions with other stressors such as environmental contaminants or other parasites [31]–[35]. Although biological mechanisms underlying relationships among stressors of honey bees are not well understood, it is likely that exploitative competition for limited resources, as well as host stress resulting from tissue pathology and immune suppression, play important roles [14], [31], [33], and could lead to numerical (i.e., intensity) or functional (i.e., realised niche) responses by parasites that are either symmetrical (both species experience equal responses) or asymmetrical [36].

It is rare for multiple microsporidian species to be parasitic within sympatric individuals of the same insect species [37]. Nonetheless, sympatric honey bee populations, and even individuals, can be co-parasitized by both *N. ceranae* and *Nosema apis* [38], [39], the latter being the historical microsporidian species of honey bees [12], [24], [40]. Similar to *N. ceranae*, *N. apis* can cause significant tissue damage in the gut that ultimately results in increased winter colony mortality or poor build-up of surviving colonies in spring [40]. Within the last decade, *N. ceranae* has been detected on all continents where honey bees are maintained, while the occurrence of *N. apis* has diminished [10], [12], [24], [41]–[43], suggesting a numerical response by *N. apis* to co-infection that has resulted in decreased prevalence and distribution of the parasite. This apparent exclusion appears to be geographically heterogeneous, and is likely governed by previously discussed genetic and environmental factors influencing dispersal and competition for limited resources during density-dependent parasite regulation [15], [18], [26]–[30], [44].

Few studies have investigated host honey bee responses to both *Nosema* parasites simultaneously or parasite reproduction under experimental conditions. Paxton et al. [45] observed higher mortality in *N. ceranae*-infected worker honey bees compared to those parasitized by *N. apis*, and no difference in spore intensity (number of vegetative parasite cells per host) between the two species. Forsgren and Fries [46] similarly found no difference in spore intensity between *N. ceranae* and *N. apis*, but they did not observea difference in mortality between workers infected by either *N. apis* or *N. ceranae*. Furthermore, using molecular techniques they did not detect a competitive advantage during co-infection by either parasite congener. Lastly, Martín-Hernández et al. [47] reported higher mortality and increased nutritional demand by workers infected with *N. ceranae* compared to *N. apis*, whereas Huang and Solter [48] reported consistently higher spore production by *N. ceranae*.

Because of the conflicting results regarding differences in host mortality caused by *N. ceranae* and *N. apis*, and because the former has only recently spread from Asia to become a global concern, comparative studies focusing on these congeneric parasites are of significant interest. Here we present an experiment that compared host mortality and nutritional demand, as well as parasite reproduction (quantified by spore intensity and DNA amount) and interspecific interactions, using honey bees artificially infected by *N. apis*, *N. ceranae*, or both. Uniquely, experimental *Nosema* and honey bees were obtained from outside of Europe, and experimental hosts were observed for over four weeks, the typical length of time that worker honey bees spend performing intra-hive duties [49]. Previous work used European-collected parasites and hosts, and terminated experiments between days 7 and 15 post inoculation. Based on laboratory and field investigations previously discussed, we hypothesised that *Nosema*-infected honey bees, in particular those parasitized by *N. ceranae*, would exhibit greater mortality than controls. We also predicted greater *N. ceranae* reproduction compared to *N. apis*. This could help to explain apparent exclusion of *N. apis* by *N. ceranae* in many regions of the world [41].

## Materials and Methods

### Experimental design

Laboratory experiments consisted of four treatment groups (1. control, 2. *N. apis*, 3. *N. ceranae*, and 4. *N. apis/N. ceranae* (hereafter, mixed)) housed at Acadia University in Wolfville, Nova Scotia, Canada. Each treatment group had 60 Buckfast honey bee workers housed in hoarding cages (wooden frame with hardware cloth and plexiglass sides; volume = 2,652 cm^3^; 20 workers per cage) in a growth chamber maintained at 33°C, ∼45% relative humidity, and in complete darkness [50].

Combs of similarly aged pupae obtained from two Buckfast colonies were used to collect workers for the experiment. In the laboratory, emerging individuals were randomly assigned to one of four treatment groups, and orally inoculated with 5 µl 75% (weight/volume) sucrose solution within 48 h of emergence. Inoculum for each worker belonging to the *Nosema* treatments contained a total of 35,000 freshly obtained local spores of the respective parasite species, enough to ensure 100% infection [46]; the mixed inoculum contained equal parts *N. apis* and *N. ceranae*. *Nosema* species confirmation was performed molecularly as described below and in Burgher-MacLellan et al. [38]. Post-inoculation, workers were group fed 50% (w/v) sucrose solution *ad libitum* for the duration of the experiment using a 10-ml syringe with the adaptor removed. The experiment was terminated at 30 d when no living workers remained for one of the treatment groups because they had either died in the cage or had been removed to quantify *Nosema* infection.

### Host mortality and food consumption

Mortality was recorded daily; dead individuals were removed from cages and stored at −80°C for later *Nosema* sp. quantification (see below). Food consumption was also measured daily to quantify nutritional demand [16] by visually recording quantities of sucrose solution depleted from syringes; per worker daily consumption was calculated by using the number of living workers at the end of each 24-h interval. Food was replaced every week to limit microbial growth and to ensure sucrose solution was provided *ad libitum* [51]. Comparison of food consumption among groups continued only until 25 d post inoculation, when one cage contained a single living worker.

### Parasite reproduction

*Nosema* spores (spores per bee) and DNA were quantified on all workers that died between 28 and 30 d (*n* = 2, 8, 7, and 9 for control, *N. apis*, *N. ceranae*, and mixed treatments, respectively) immediately prior to experiment termination. Spores were further quantified at 7, 14, and 21 d post inoculation using three randomly chosen living workers per treatment (one per cage). All workers were stored immediately at −80°C after collection from cages until laboratory analyses.

### *Nosema* quantification – microscopy

For each individual honey bee, suspensions were created by crushing its abdomen with a pellet pestle in 1 ml distilled water. *Nosema* spores were counted in these suspensions using a haemocytometer and light microscopy (Thermo Fisher Scientific, Waltham, Massachusetts, USA) [52], [53].

### *Nosema* quantification - simplex real-time PCR

*Nosema* DNA (ng) was quantified using methods outlined by Burgher-MacLellan et al. [38]. This included use of primer pairs 218MITOC (*N. ceranae*) and 321APIS (*N. apis*) that were originally optimized by Martin-Hernandez *et al.* [54], as well as qPCR methods developed by Forsgren and Fries [46] that applied external DNA standards of serial diluted PCR amplicons. Briefly, genomic DNA was isolated from each honey bee by pre-treating a 250-µl aliquot of a crushed abdomen suspension (described in the previous section) with 10 µl proteinase K (20 mg/ml) (Sigma-Aldrich Canada, Oakville, Ontario, Canada) for 20 min at 37°C. DNA was then purified using a modified protocol (steps 1–3 omitted) from the Ultra Clean Tissue DNA Extraction Kit (Mo Bio Laboratories, Carlsbad, California, USA). DNA was quantified using a Nanodrop 1000 spectrophotometer (Fisher Scientific, Ottawa, Ontario, Canada), and samples stored at −20°C until real-time PCR was performed.

Simplex quantitative real-time PCR (qPCR) was performed using an Mx4000 thermocycler (Stratagene, La Jolla, California, USA). Each separate qPCR reaction consisted of 12.5 µl Maxima SYBR Green/Rox qPCR master mix (Thermo Scientific, Rockford, Illinois, USA), 0.2 µl *N. apis* or *N. ceranae* primer sets [54], 1 µl template (100 ng genomicDNA) and nuclease-free water to a final volume of 25 µl. For each primer pair, the PCR reactions were performed in triplicate on the same plate and contained negative and positive controls (no template DNA and DNA isolated from *N. apis* or *N. ceranae* spores). Triplicate means were reported. PCR amplification parameters included an initial 10-min denaturing period at 95°C followed by 40 cycles of 30-s denaturing at 95°C, 30-s annealing at 60°C, and 30-s extension at 72°C, and a final 5-min extension period at 72°C. Amplified products were confirmed using melting curve analysis plots where temperature profiles were 1 min at 95°C, 30 s at 55°C, followed by forty 30-s increases of 1°C, and a final holding temperature at 4°C. Each simplex qPCR run included the appropriate quantification standard curve (*i.e. R^2^*>0.98 and primer efficiency >94%) prepared using serial dilutions (1.0^−1^ to 1.0^−7^) ng of purified PCR products (*N. apis* and *N. ceranae*) for target DNA. Bee DNA samples were quantified for *Nosema* DNA amount by plotting cycle threshold (Ct) values against nanograms of target DNA.

### Statistical analyses

All statistical analyses were performed using R 2.15.2 (R Development Core Team; Vienna, Austria), except for the survival analysis which was performed using Minitab 16 (Minitab Inc., State College, Pennsylvania, USA). Cumulative mortality was analysed using the Kaplan-Meier Log-Rank survival analysis for ‘censored’ data because time of death for some workers was not known (i.e., some living workers were killed periodically to quantify spore intensity during the experiment, and some were still living when the experiment was terminated) [55]. Food consumption and *Nosema* intensities were evaluated using ANOVAs or Repeated Measures ANOVAs; Tukey's HSD post hoc tests were used for multiple comparisons among treatments. Where appropriate, data were square-root transformed to improve fit to normality.

## Results

### Host mortality and food consumption

**Figure 1:**
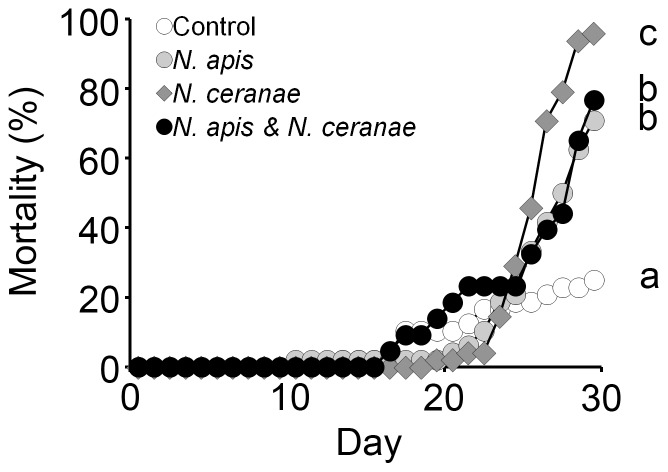
Effect of *Nosema* infection on mortality of adult worker western honey bees (*Apis mellifera*). Mortality is shown as the cumulative percentage of dead individuals from control, *Nosema apis*, *Nosema ceranae* and mixed *N. apis*/*N. ceranae* treatments each day. The experiment was terminated at 30 d post inoculation. Treatments with different letters had significant differences in mortality.

Mortality at 30 d post-inoculation was 25.0, 70.0, 95.0, and 76.7% for control, *N. apis*, *N. ceranae*, and mixed treatments, respectively (Fig. 1). Workers in the *N. ceranae* treatment had significantly increased mortality compared to workers from the other treatments (Kaplan-Meier Log-Rank Test, all *P*s<0.002), whereas controls had significantly lower mortality compared to all other treatments (Kaplan-Meier Log-Rank, all *P*s<0.001). Mortality did not differ significantly between workers in the *N. apis* and mixed treatments (Kaplan-Meier Log-Rank, *P* = 0.67) (Fig. 1).

**Figure 2:**
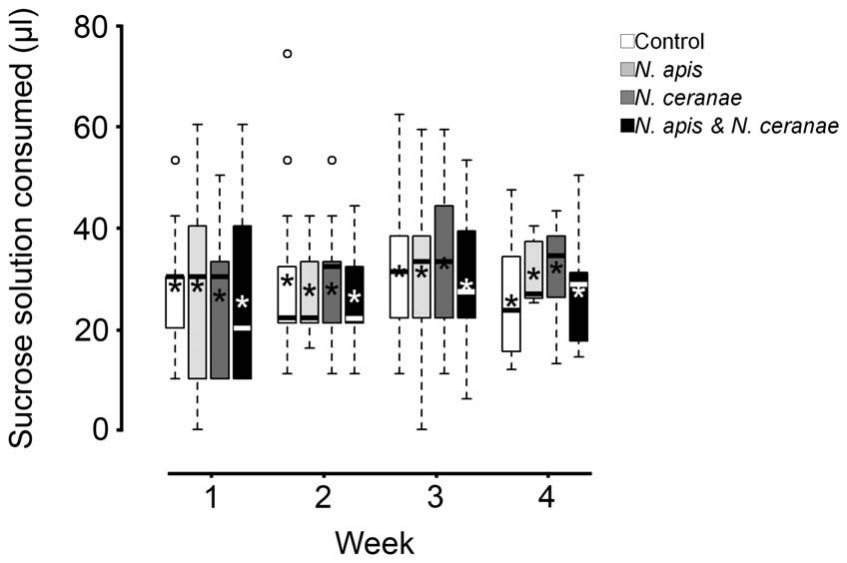
Effect of *Nosema* infection on adult worker western honey bee (*Apis mellifera*) nutritional demand. Consumption is shown as volume of 50% (weight/volume) sucrose-water mixture per bee per week post inoculation for control, *Nosema apis*, *Nosema ceranae*, and mixed *N. apis*/*N. ceranae* treatments (Week 4 included only consumption from 22–25 d post inoculation). Boxplots show interquartile range (box), median (black or white line within interquartile range), data range (dashed vertical lines), and outliers (open dots); asterisks (black or white) represent means. No significant differences were observed among treatments for daily consumption per worker.

No difference was observed among treatments in food consumption (Repeated Measures ANOVA, *F*_3,8_ = 0.4, *P* = 0.79) (Fig. 2).

### Parasite reproduction

**Figure 3:**
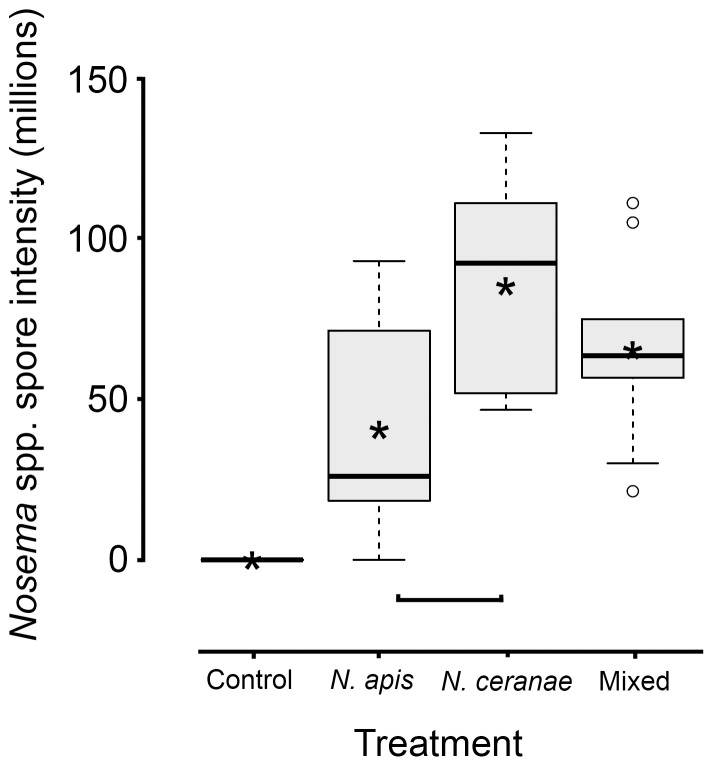
Level of *Nosema* infection in dead adult worker western honey bees (*Apis mellifera*) 28–30 d post oral inoculation for control, *Nosema apis*, *Nosema ceranae* and mixed *N. apis*/*N. ceranae* treatments. Boxplots show interquartile range (box), median (black line within interquartile range), data range (dashed vertical lines), and outliers (open dots); asterisks (black) represent means. Horizontal square parenthesis under boxplots indicates a significant difference; controls were excluded from analyses because no infections were observed.

**Figure 4:**
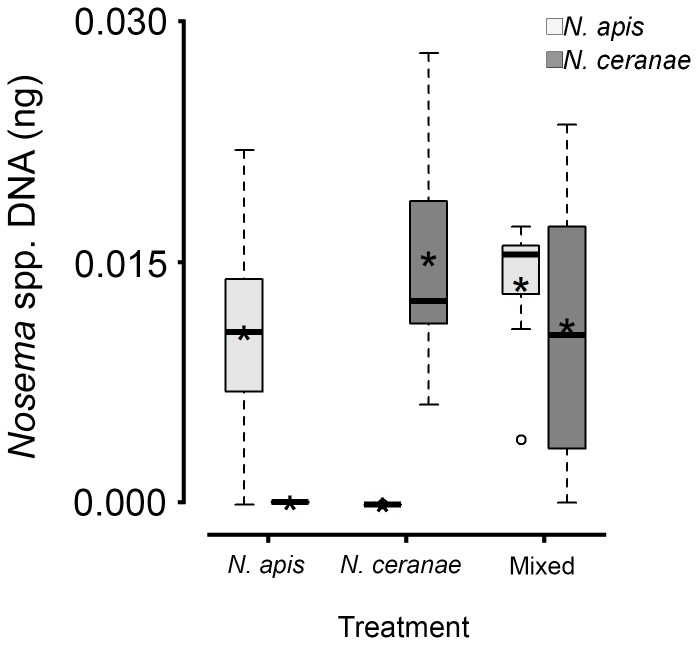
Levels of *Nosema apis* and *Nosema ceranae* DNA (square root-transformed) in adult worker western honey bees (*Apis mellifera*) that died between 28 and 30 d post inoculation in *N. apis*, *N. ceranae* or mixed *N. apis*/*N. ceranae* treatments (same workers shown in **Fig. 3**). Boxplots show interquartile range (box), median (black or white line within interquartile range), data range (dashed vertical lines), and outliers (open dots); asterisks (black or white) represent means. No significant differences were observed in quantities among the four instances where we expected to find DNA (i.e., the boxes with means above 0).

**Figure 5:**
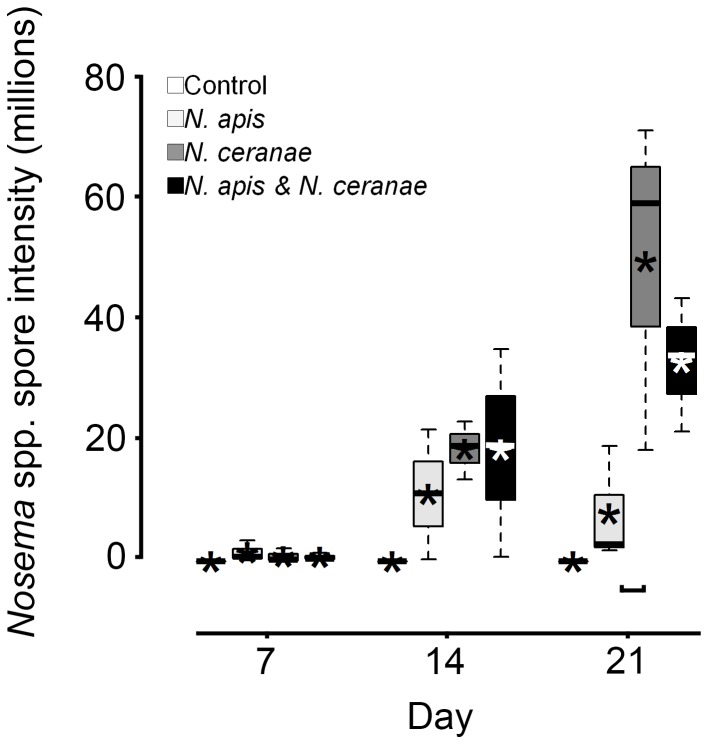
*Nosema* infection intensities in live-sampled adult worker western honey bees (*Apis mellifera*) at 7, 14, and 21 d post oral inoculation in control, *Nosema apis*, *Nosema ceranae*, and mixed *N. apis*/*N. ceranae* treatments. Boxplots show interquartile range (box), median (black or white line within interquartile range), data range (dashed vertical lines), and outliers (open dots); asterisks (black or white) represent means. Horizontal square parenthesis under boxplots indicates a significant difference; controls were excluded from analyses because no infections were observed.

In workers that died between 28 and 30 d post inoculation, *Nosema* spore intensities were significantly different among groups (Fig. 3). Spore intensity in *N. ceranae* workers was greater than in *N. apis* workers (Tukey's HSD, adjusted *P* = 0.03), but not compared to workers from the mixed group (Tukey's HSD, adjusted *P* = 0.60). No difference in spore intensity was observed between workers from the *N. apis* and mixed treatments (Tukey's HSD, adjusted *P* = 0.16) (Fig. 3). Additionally, no difference in the quantity of *N. apis* DNA was observed between *N. apis* and mixed treatments (all Tukey's HSD, adjusted *P*≥0.50), or of *N. ceranae* DNA quantity between *N. ceranae* and mixed treatments (all Tukey's HSD, adjusted *P*≥0.62) (Fig. 4). Despite greater spore intensities for *N. apis* and *N. ceranae* treatments at 7 and 14 d in live sampled workers, respectively, no statistical differences were observed (both ANOVAs, *F*_2,6_≤0.5, *P*s≥0.62; Fig. 5). At 21 d, however, spore intensity was significantly greater in the *N. ceranae* than in the *N. apis* treatment (Tukey's HSD, *P* = 0.05).

## Discussion

Our experiment demonstrated that *Nosema* infection significantly increased honey bee worker mortality but had no influence on food consumption. Spore intensity and mortality was significantly greater for *N. ceranae*-infected individuals compared to those infected by *N. apis*. This supports claims that *N. ceranae* could be one stressor responsible for elevated colony losses that have been observed recently [3], [4], [20], [23], and suggests that high spore production could be a mechanism by which apparent rapid range extension of this horizontally-introduced species occurred.

To our knowledge this is the first laboratory study to follow simultaneously both *N. apis* and *N. ceranae* intensity beyond two weeks to measure effects on hosts using species from outside of Europe [45]–[47]. Length of observation is particularly important because mean honey bee worker longevity during the foraging season (when this study was performed) is between 15 and 60 d [49]. *Nosema* spore intensities in workers that died between 28 and 30 d post-inoculation were consistent with spore intensity data collected from live workers at 21 d post inoculation, wherein *N. ceranae* reproduction was significantly greater than that of *N. apis*. Conversely, quantity of *Nosema* DNA did not differ between congeners. It is likely that *Nosema* DNA that we detected represented immature stages within host cells rather than mature spores due to a dense wall surrounding each spore [40], [56]. Spore dimorphism (thin-walled spores germinate within hosts whereas thick-walled spores are released into the environment) are known from the family Nosematidae, including *N. apis* [40]. It is possible that higher spore intensity of *N. ceranae* compared to *N. apis* is the result of a faster multiplication rate and greater investment in environmentally resistant spores that do not reinfect gut epithelial cells, but rather reside in the rectum until they are released into the environment via contaminated faeces [48]. Unfortunately, little is known about the biology, including life cycle and spore production, of *N. ceranae* in honey bees. Greater potential for faecal-oral horizontal transmission resulting from high levels of *N. ceranae* spores in the environment could explain why the distribution of *N. ceranae* has increased rapidly in recent years, and why the parasite can be found in contaminated materials in the hive or on forage [57], [58].

Based on spore intensity, it appears that carrying capacity within honey bees, or at least maximum population size, can be much greater for *N. ceranae* than for *N. apis*. Despite our extended observation of workers, neither our data nor those of previous studies that observed spore intensities regularly for shorter time periods obtained asymptotic *N. ceranae* intensities [15], [45]. It is possible that smaller spore size [59], broader tissue tropism [56], and limited time for co-evolution [36], at least compared to *N. apis*, could help to explain this.

Results from mixed infections suggested competition between *N. apis* and *N. ceranae*. If full infection occurs regardless of initial spore inocula [60], [61], we would expect parasite intensities from the mixed treatment to be the sum of both single *Nosema* infections; this was clearly not observed because spore intensity was of intermediate intensity. Unfortunately, similar size and shape of *N. apis* and *N. ceranae* spores did not make it possible to accurately distinguish species [59] using light microscopy; therefore, we could not determine if symmetrical or asymmetrical competition occurred for a particular species. Recently, Martin-Hernandez et al. [62] demonstrated that *N. ceranae* does not replace *N. apis*, at least in Spain. This suggests that both parasites in our study could have responded equally to competition rather than asymmetrically wherein one out-competes the other. Conversely, DNA quantities in single and mixed infections did not suggest competition because no difference in parasite intensity was observed, regardless of treatment. Forsgren and Fries [46] similarly did not observe competition between *Nosema* species based on molecular methods; they did not investigate spore levels using light microscopy. This could suggest a functional response by one or both parasites, whereby host cells can be parasitized by *Nosema* equally but reproductive output (i.e., number of environmental spores) is unaffected.

We did not observe differences in energetic demand, as measured by sucrose consumption, among treatment groups. This was unexpected because parasites usually, but not always [63], compete with their hosts for nutrients [64] to increase nutritional demand. In previous studies, *Nosema*-infected workers had significantly increased demand for energy, which was also measured by carbohydrate sucrose consumption [16], [31], [47], as well as increased sugar metabolism [14]. However, not all studies have observed this phenomenon [34]. Possibly, experimental methods (e.g., spore inoculation dose, observation period, testing arena) explain these differences.

Controversy remains over the role of *Nosema* gut parasites in the recent high honey bee colony mortalities observed in many parts of North America and Europe [20]–[22], [24], [25], [32]. This could be due to both genetic and environmental (or methodological) factors. As suspected for *Nosema bombi* microsporidians in bumble bees [65], genetic variants of *Nosema* species infecting honey bees may differ in virulence [26], [66], but likewise host genetics could also affect susceptibility [15], [23]. Additionally, some commonly used agro-chemicals may interact with *N. ceranae* [31], [34], [61], and Deformed wing and Black queen cell viruses were negatively and positively correlated with *N. ceranae* and *N. apis*, respectively [33], [67]. Unfortunately, broad-scale screening for these extrinsic factors in experimental workers, as well as their source colonies, is costly and not regularly performed during standard laboratory assays. Furthermore, variation in laboratory methods employed by researchers could further contribute to our foggy understanding of these host-parasite systems as recently highlighted by Fries et al. [53] and Williams et al. [51].

Here, in a long-term laboratory cage study using parasites and hosts residing outside of Europe, we demonstrated that parasitism by *Nosema*, in particular by the invasive *N. ceranae* compared to the historic *N. apis*, increased honey bee worker mortality. We also observed higher spore intensity in honey bees parasitized by *N. ceranae* compared to *N. apis*, and a numerical response in spore production during co-infection; this is likely important to inter-host horizontal parasite transmission that relies on ingestion of spores, and that should be further investigated to better understand epidemiology of these important honey bee parasites.
